# Integration of multi-omics profiling reveals an epigenetic-based molecular classification of lung adenocarcinoma: implications for drug sensitivity and immunotherapy response prediction

**DOI:** 10.3389/fphar.2025.1540477

**Published:** 2025-02-19

**Authors:** Ning Wang, Yinan Li, Yaoyao Wang, Wenting Wang

**Affiliations:** ^1^ Department of Respiratory and Critical Medicine, Qingdao Municipal Hospital, Qingdao University, Qingdao, China; ^2^ Department of Oncology, Qingdao Municipal Hospital, University of Health and Rehabilitation Sciences, Qingdao, China

**Keywords:** lung adenocarcinoma, epigenetic regulation, molecular classification, immune microenvironment, precision medicine, machine learning, prognostic model, immunotherapy

## Abstract

**Background:**

Lung adenocarcinoma (LUAD) remains a major cause of cancer-related mortality worldwide, with high heterogeneity and poor prognosis. Epigenetic dysregulation plays a crucial role in LUAD progression, yet its potential in molecular classification and therapeutic prediction remains largely unexplored.

**Methods:**

We performed an integrated multi-omics analysis of 432 LUAD patients from TCGA and 398 patients from GEO datasets. Using consensus clustering and random survival forest (RSF) algorithms, we established an epigenetic-based molecular classification system and constructed a prognostic model. The model’s performance was validated in multiple independent cohorts, and its biological implications were investigated through comprehensive functional analyses.

**Results:**

We identified two distinct molecular subtypes (CS1 and CS2) with significant differences in epigenetic modification patterns, immune microenvironment, and clinical outcomes (P = 0.005). The RSF-based prognostic model demonstrated robust performance in both training (TCGA-LUAD) and validation (GSE72094) cohorts, with time-dependent AUC values ranging from 0.625 to 0.694. Low-risk patients exhibited enhanced immune cell infiltration, particularly CD8^+^ T cells and M1 macrophages, and showed better responses to immune checkpoint inhibitors. Drug sensitivity analysis revealed subtype-specific therapeutic vulnerabilities, with low-risk patients showing higher sensitivity to conventional chemotherapy and targeted therapy.

**Conclusion:**

Our study establishes a novel epigenetic-based classification system and predictive model for LUAD, providing valuable insights into patient stratification and personalized treatment selection. The model’s ability to predict immunotherapy response and drug sensitivity offers practical guidance for clinical decision-making, potentially improving patient outcomes through precision medicine approaches.

## 1 Introduction

Lung cancer remains the leading cause of cancer-related deaths globally. It primarily manifests in two forms: Small Cell Lung Cancer and Non-Small Cell Lung Cancer (NSCLC), with NSCLC accounting for approximately 85% of all lung cancer cases and demonstrating a mere 26% 5-year survival rate. NSCLC predominantly comprises Lung Adenocarcinoma (LUAD) and squamous cell carcinoma, with LUAD representing approximately 70% of all NSCLC cases and exhibiting poor prognosis (Niu et al., 2022). The diagnosis and treatment of LUAD face several critical challenges: the absence of early symptoms often results in late-stage diagnosis; high tumor heterogeneity complicates personalized treatment approaches; and poor drug tolerance and resistance development significantly impact treatment efficacy (Cheng Y. et al., 2021; Wu and Lin, 2022). Currently, standard LUAD treatment protocols primarily encompass surgical resection, radiotherapy, chemotherapy, and immune checkpoint inhibitor therapy (Sun et al., 2024; Passaro et al., 2022). However, these conventional therapeutic approaches present significant limitations: surgery is only viable for early-stage patients; radio- and chemotherapy often induce severe adverse effects with limited efficacy; and immunotherapy demonstrates variable response rates while carrying risks of immune-related adverse events (Cheng Y. et al., 2021; Wang J. et al., 2021). Consequently, identifying LUAD-associated biomarkers and exploring novel therapeutic strategies have become focal points in current clinical research.

In recent years, epigenetic therapy has garnered substantial attention. Epigenetic modifications, including DNA methylation, histone modifications, and chromatin remodeling, serve as crucial molecular switches that dynamically regulate gene expression patterns without altering the underlying DNA sequence. In normal cells, these epigenetic mechanisms precisely control spatiotemporal gene expression to maintain cellular homeostasis (Chen et al., 2020). Previous studies on the molecular subtyping of LUAD have mainly focused on genomic changes, with relatively limited attention paid to epigenetic mechanisms. At present, some studies have explored subtypes based on DNA methylation in LUAD. For example, Zhao et al. identified two subtypes associated with LUAD prognosis through DNA methylation typing (Zhao et al., 2021). However, these studies usually examine DNA methylation alone, which may miss important biological interactions. Compared with genomic profiling, epigenetic-based classification has unique advantages: it better reflects the dynamic nature of cancer progression, shows stronger correlation with treatment response, and can capture regulatory mechanisms that may be missed through genomic analysis alone. Recent studies have demonstrated that dysregulation of these epigenetic mechanisms significantly promotes LUAD initiation, progression, and therapeutic resistance (Fan et al., 2024). For instance, Rowbotham et al. demonstrated that H3K9 methyltransferases and demethylases control lung tumor proliferating cells and cancer progression by regulating extracellular matrix genes through G9a suppression, driving lung adenocarcinoma cells toward the TPC phenotype (Rowbotham et al., 2018). Li et al.'s research revealed that histone demethylases (such as JARID1B and LSD1) influence chromatin structure and gene expression by removing histone methyl modifications (Li et al., 2011). Bajbouj et al. reported the potential role of histone modifications in NSCLC treatment, noting that epigenetic alterations in H2A (H2AK5ac) and H3 (H3K4me2, H3K9ac) possess higher prognostic value in early-stage NSCLC (Bajbouj et al., 2021). Furthermore, these epigenetic alterations can modulate the tumor microenvironment and influence immune surveillance mechanisms, indicating their potential as therapeutic targets (Hogg et al., 2020). Epigenetic therapy offers unique advantages compared to other treatments: reversibility through pharmaceutical intervention; tissue and cell specificity enabling precise treatment; and the ability to enhance immunotherapy efficacy while reversing tumor drug resistance (Yu et al., 2024; Topper et al., 2020).

In this study, we proposed an integrated approach to identify clinically relevant molecular subtypes in LUAD by leveraging single-cell sequencing technology and advanced machine learning algorithms in combination with epigenetic and transcriptomic data, with the primary goal of improving treatment stratification and patient outcomes (Baysoy et al., 2023). Our specific objectives were to: establish robust LUAD molecular subtypes based on integrated epigenetic and transcriptomic signatures to effectively guide clinical decisions; develop and validate a practical classification model that can be easily implemented in a clinical setting for patient stratification; and evaluate how these subtypes can inform treatment selection, particularly for immunotherapy and targeted therapies. This integrated approach addresses a critical gap in the current management of LUAD by providing a more comprehensive molecular classification system that is directly relevant to treatment decisions. For example, identifying subtypes with distinct immunological signatures can help select patients who are more likely to respond to immunotherapy, while understanding epigenetic patterns associated with drug sensitivity can guide the selection of targeted therapies. Such stratification is critical to advancing precision medicine for the treatment of LUAD, with the potential to improve response rates and patient outcomes while reducing unnecessary treatments and associated costs.

## 2 Materials and methods

### 2.1 Data source

This study primarily analyzed two large Lung Adenocarcinoma (LUAD) cohorts. The main analysis cohort was derived from The Cancer Genome Atlas (TCGA-LUAD, https://portal.gdc.cancer.gov/), comprising multi-omics data from 432 patients, including mRNA expression profiles, miRNA expression profiles, long non-coding RNA (lncRNA) expression profiles, DNA methylation profiles, and somatic mutation information (Tomczak et al., 2015). The first validation cohort, GSE72094, was obtained from the Gene Expression Omnibus (GEO, https://www.ncbi.nlm.nih.gov/geo/) database, containing gene expression profiles and clinical follow-up data for 398 LUAD patients.

To validate the model’s predictive value in immunotherapy, we incorporated two additional Non-Small Cell Lung Cancer (NSCLC) cohorts that received immune checkpoint inhibitor therapy: GSE91061 (109 patients receiving anti-PD-1/CTLA4 treatment) and GSE135222 (27 patients receiving anti-PD-1 treatment). All gene expression data underwent standardization to eliminate batch effects. The epigenetic regulatory gene set was sourced from the EpiFactors database (http://epifactors.autosome.ru/), which systematically catalogs human protein complexes associated with epigenetic modifications (Marakulina et al., 2023).

To address potential batch effects between different data sources, we implemented a systematic data harmonization strategy. Raw data from both TCGA and GEO datasets underwent consistent preprocessing: (1) probe-level data were mapped to gene symbols using manufacturer-provided annotation files; (2) when multiple probes mapped to the same gene, the probe with the highest mean intensity was retained; (3) missing values were imputed using k-nearest neighbor algorithm (k = 10).

For batch effect correction, we employed a two-step approach: (1) ComBat algorithm from the sva R package was applied to remove systematic batch effects while preserving biological variations; (2) quantile normalization was performed to ensure comparable distribution of expression values across datasets. The effectiveness of batch correction was evaluated through principal component analysis (PCA) and relative log expression (RLE) plots before and after correction. Additionally, we performed correlation analysis between technical replicates across different platforms to ensure data consistency. These procedures effectively minimized technical variations while maintaining biological signals, enabling reliable integration of multi-source data for downstream analyses.

All analyses were performed using R version 4.4.0.

### 2.2 Molecular subtype characterization through multi-omics data integration

To identify LUAD molecular subtypes, we employed the MOVICS algorithm for integrated multi-omics clustering analysis (Lu et al., 2021). The MOVICS package was implemented using a multi-step approach (Version: 0.99.17). For feature selection, we first filtered epigenetics-related genes and performed survival analysis (Cox regression, p < 0.05) on mRNA expression data. For other molecular features, we applied the following criteria: top 1500 MAD-filtered lncRNAs followed by survival filtering (p < 0.05); top 50% MAD-filtered miRNAs with survival significance (p < 0.05); top 1500 MAD-filtered methylation sites with survival significance (p < 0.05); and mutation features present in >5% of samples. The optimal cluster number was determined by testing k = 2-8 using multiple clustering methods. Integration was performed using Gaussian models for expression and methylation data, and binomial model for mutation data. Clustering robustness was assessed using silhouette analysis and consensus clustering with euclidean distance and average linkage. Data standardization employed centerFlag and scaleFlag parameters for expression and methylation features, with methylation values converted to M-values for enhanced signal detection.

Initially, we conducted feature selection for each data type: for mRNA expression, we focused on epigenetic-related genes and selected survival-associated features using Cox regression (p < 0.05). For lncRNA and methylation data, we initially selected 1,500 features with the highest variation using Median Absolute Deviation (MAD), followed by survival-based screening (Cox p < 0.05). For miRNA expression, we retained the top 50% features by variation and further filtered them through Cox regression (p < 0.05). For mutation data, we selected genes with mutation frequencies exceeding 5%. The optimal cluster number was determined through multiple clustering evaluation metrics. Subsequently, we applied a multi-omics integration clustering method that combined Gaussian distribution models for expression and methylation data with binomial distribution models for mutation data. Clustering robustness was evaluated through consensus clustering and silhouette analysis by using ConcensusClusterPlus package (Version: 1.66.0) (Wilkerson and Hayes, 2010). To visualize molecular subtypes, we generated comprehensive heatmaps displaying patterns of selected features across different omics levels. Survival differences between identified subtypes were assessed using Kaplan-Meier analysis.

### 2.3 Transcriptional regulation and immune microenvironment characteristics of LUAD molecular subtypes

Building upon the molecular subtyping results, we further explored the biological characteristics of different molecular subtypes. Initially, we selected key transcription factors including FOXM1, EGFR, KLF4, and epigenetic regulatory genes such as SIRT6 and EHMT2 to construct transcriptional regulatory networks using the RTN algorithm (Dai et al., 2020), evaluating their activity differences across subtypes. Subsequently, we employed multiple methods to assess tumor immune microenvironment characteristics: quantifying tumor-infiltrating lymphocyte levels using MeTIL scores (Zou et al., 2021), evaluating tumor purity, stromal and immune cell infiltration using the ESTIMATE algorithm (Yoshihara et al., 2013), analyzing expression profiles of immune checkpoint-related genes including PD-1/PD-L1, and deconvoluting the composition of 22 immune cell types using the CIBERSORT algorithm (Guan et al., 2022; Chen et al., 2018). Finally, to verify the stability and reproducibility of molecular subtyping, we constructed an NTP classifier based on differential genes and employed PAM algorithm for cross-validation (Yoshihara et al., 2013), validating the classification results in an independent cohort (GSE72094) while assessing consistency between different classification methods.

### 2.4 Performance evaluation of integrated machine learning models in LUAD prognosis prediction

Based on the preceding multi-omics molecular subtyping results, we constructed various machine learning prognostic prediction models. Using TCGA-LUAD as the training set and GSE72094 as the independent validation set, we first performed standardized data preprocessing. We then implemented multiple baseline machine learning algorithms, including Random Survival Forest (RSF) (Becker et al., 2023), Elastic Net, stepwise regression for Cox proportional hazards model (StepCox), CoxBoost, partial least squares regression (plsRcox), principal component analysis (SuperPC), Gradient Boosting Machine (GBM), and Support Vector Machine (survival-SVM). Additionally, we explored ensemble learning strategies combining various feature selection methods with algorithms, such as combinations of RSF, Lasso, StepCox, and CoxBoost feature selection with other algorithms. Using C-index as the evaluation metric, we visualized and compared the predictive performance of different models across datasets through heatmaps, analyzing performance differences between single algorithms and ensemble strategies, as well as model stability across training and validation sets.

### 2.5 Validation and in-depth analysis of machine learning prognostic models

Based on the model comparison results, we selected the best-performing RSF model for detailed analysis with package of randomForestSRC (Version: 3.3.1). Initially, we employed Variable Importance Analysis (VIMP) to evaluate each gene’s contribution to prognosis prediction, visualizing the top 20 genes with the highest importance scores. Subsequently, we constructed a risk prediction model based on these key genes through the following process: (1) z-score standardization of gene expression data; (2) utilization of RSF algorithm mortality predictions as risk scores; (3) determination of optimal risk grouping thresholds by maximizing log-rank test statistics. To evaluate model predictive performance, we conducted time-dependent ROC curve analysis with survival package (Version: 3.5.8) for 1-year, 3-year, and 5-year prognostic predictions, with quantitative assessment through AUC values by using timeROC package (Version: 0.4). Simultaneously, we employed Kaplan-Meier survival analysis and log-rank tests to evaluate survival differences between high- and low-risk groups. All analyses were performed in both TCGA training and GSE72094 validation sets to verify model stability and reproducibility.

### 2.6 Multi-dimensional clinical feature validation of risk prediction model

To comprehensively evaluate the clinical utility of the RSF risk prediction model, we conducted multi-layered validation analyses. We initially employed pie charts to visualize the distribution differences of clinical features between high- and low-risk groups, including TNM staging, clinical staging, and gender, with chi-square tests assessing statistical significance. Subsequently, we analyzed risk score distributions across different T stages using violin plots and box plots combined with Wilcoxon rank-sum tests. Concurrently, we constructed heatmaps featuring model-selected marker genes, demonstrating their expression patterns across risk groups and clinical phenotypes. Additionally, we employed ROC curves to assess the model’s stratification capability between early and late-stage patients (Stage I + II vs. III + IV). Finally, we conducted survival analyses within clinical stage subgroups and age subgroups to validate the model’s prognostic prediction value in early-stage patients. All visualizations were implemented using R software packages including pheatmap, ggplot2, and survminer.

### 2.7 Independent prognostic value assessment and nomogram construction for survival prediction model

To evaluate the independent prognostic value and clinical application potential of the risk prediction model, we conducted systematic statistical analyses. Initially, we assessed the association between prognostic factors (age, gender, TNM staging, clinical staging, and risk scores) and survival outcomes through univariate Cox regression analysis, visualizing hazard ratios (HR) and their 95% confidence intervals through forest plots. Subsequently, statistically significant factors were incorporated into a multivariate Cox regression model to validate the independent prognostic value of the risk score. Based on the multivariate Cox model, we constructed nomograms integrating clinicopathological features and evaluated the accuracy of 1-year, 3-year, and 5-year survival predictions through calibration curves. Furthermore, we employed Decision Curve Analysis (DCA) to assess the model’s clinical decision-making value (Rousson and Zumbrunn, 2011) and compared the discriminative ability of different predictive factors through time-dependent C-index. All statistical analyses were implemented using R software packages including rms, timeROC, and survcomp, with p < 0.05 considered statistically significant.

### 2.8 Functional annotation and pathway enrichment analysis of risk score model

To investigate the biological mechanisms reflected by the risk score, we conducted systematic functional enrichment analyses (Ashburner et al., 2000; Ogata et al., 1999). Initially, we performed differential expression analysis between high- and low-risk groups using the limma package to identify significantly differentially expressed genes. Subsequently, we employed the Gene Set Variation Analysis (GSVA) algorithm to assess Hallmark gene set activity levels in each sample. GSVA scores underwent intergroup differential analysis, with t-tests identifying significantly altered signaling pathways. We utilized the corrplot package to generate correlation heatmaps between risk scores and pathway activities, revealing key regulatory networks. Furthermore, we stratified samples into high and low expression groups based on pathway activity medians, evaluating the association between important pathways and prognosis through Kaplan-Meier survival analysis and Cox proportional hazards regression. All analyses were implemented using R software packages including GSVA, limma, and survminer, with statistical significance set at p < 0.05 after multiple testing correction.

### 2.9 Analysis of immune microenvironment features and Their Association with risk scores

To comprehensively decipher the relationship between risk scores and tumor immune microenvironment, we conducted multi-level immunological feature analyses with IOBR package (Version: 0.99.0). We initially calculated stromal scores, immune scores, and ESTIMATE scores for each sample using the ESTIMATE algorithm, comparing differences between high- and low-risk groups. ESTIMATE was selected for its validated ability to quantify tumor purity and stromal/immune cell infiltration in bulk transcriptome data. Subsequently, we evaluated immune function and immune cell activity using the ssGSEA algorithm based on predefined immune-related pathway gene sets (Lin et al., 2021), visualizing immune characteristic patterns across different risk groups through heatmaps. Furthermore, we employed the CIBERSORT algorithm to infer the proportions of 22 immune cell types, demonstrating immune cell infiltration differences between high- and low-risk groups through violin plots. CIBERSORT was chosen as our primary method for immune cell deconvolution due to its superior performance in LUAD benchmarking studies and ability to resolve 22 immune cell types. Finally, we assessed correlations between risk scores and various immune cell contents through Spearman correlation analysis (Eden et al., 2022), visualizing correlation strength and statistical significance through bubble plots. All intergroup comparisons utilized Wilcoxon rank-sum tests, while correlation analyses employed Spearman rank correlation, with p < 0.05 considered statistically significant. All analyses were implemented using R software packages including IOBR, GSVA, and ggplot2. While these methods have inherent limitations in detecting rare cell populations (abundance <5%) and tumors may be affected by this limitation, these challenges were addressed through our estimate-based normalization and stringent quality control (inverse tumor p-value <0.05).

### 2.10 Immunotherapy response prediction and immune function assessment

To validate the RSF model’s predictive value for immunotherapy response, we conducted systematic validation across multiple independent cohorts. We initially evaluated the association between risk scores and treatment response in the IMvigor210 immunotherapy cohort, analyzing both 6-month and 12-month survival outcomes, as well as the relationship between treatment response (CR/PR/SD/PD) and risk scores. Subsequently, we employed multiple computational methods to assess immune function characteristics: utilizing the Tracking Tumor Immunophenotype (TIP) algorithm to evaluate tumor immune phenotypes and calculate different immune cell infiltration levels (Xu et al., 2018); applying the Tumor Immune Dysfunction and Exclusion (TIDE) algorithm to predict immune checkpoint inhibitor treatment response (Jiang et al., 2018). Through the SubMap algorithm, we analyzed the consistency between our classification system and published immunotherapy-related datasets (GSE91061) (Shen et al., 2020), evaluating the correspondence between high/low-risk groups and immunotherapy response/non-response groups. Finally, we conducted independent validation in GSE135222 and GSE91061 cohorts. All analyses were implemented using R software packages including survminer and ComplexHeatmap, with intergroup comparisons utilizing Wilcoxon tests and survival analyses employing log-rank tests, considering p < 0.05 statistically significant.

### 2.11 Drug sensitivity prediction analysis

To explore the risk score model’s predictive value for chemotherapy drug sensitivity, we conducted systematic drug response prediction analysis using the pRRophetic package (Version: 0.5) (Geeleher et al., 2014). Initially, we constructed drug response prediction models based on drug sensitivity data and gene expression profiles from the Cancer Genome Project (CGP) 2016 database. For each sample in the TCGA-LUAD cohort, we predicted IC50 values for all available drugs in the CGP database (Sebaugh, 2011). We compared drug sensitivity differences between high- and low-risk groups using Wilcoxon rank-sum tests and visualized significantly different drugs (p < 0.05) through box plots. To ensure result reliability, error catching and handling were implemented for each drug’s prediction process. Finally, we ranked and output the analysis results for all drugs, focusing on potential therapeutic drugs demonstrating significant sensitivity differences between high- and low-risk groups. All analyses were implemented using R software packages including pRRophetic, ggplot2, and rstatix.

## 3 Results

**FIGURE 1:**
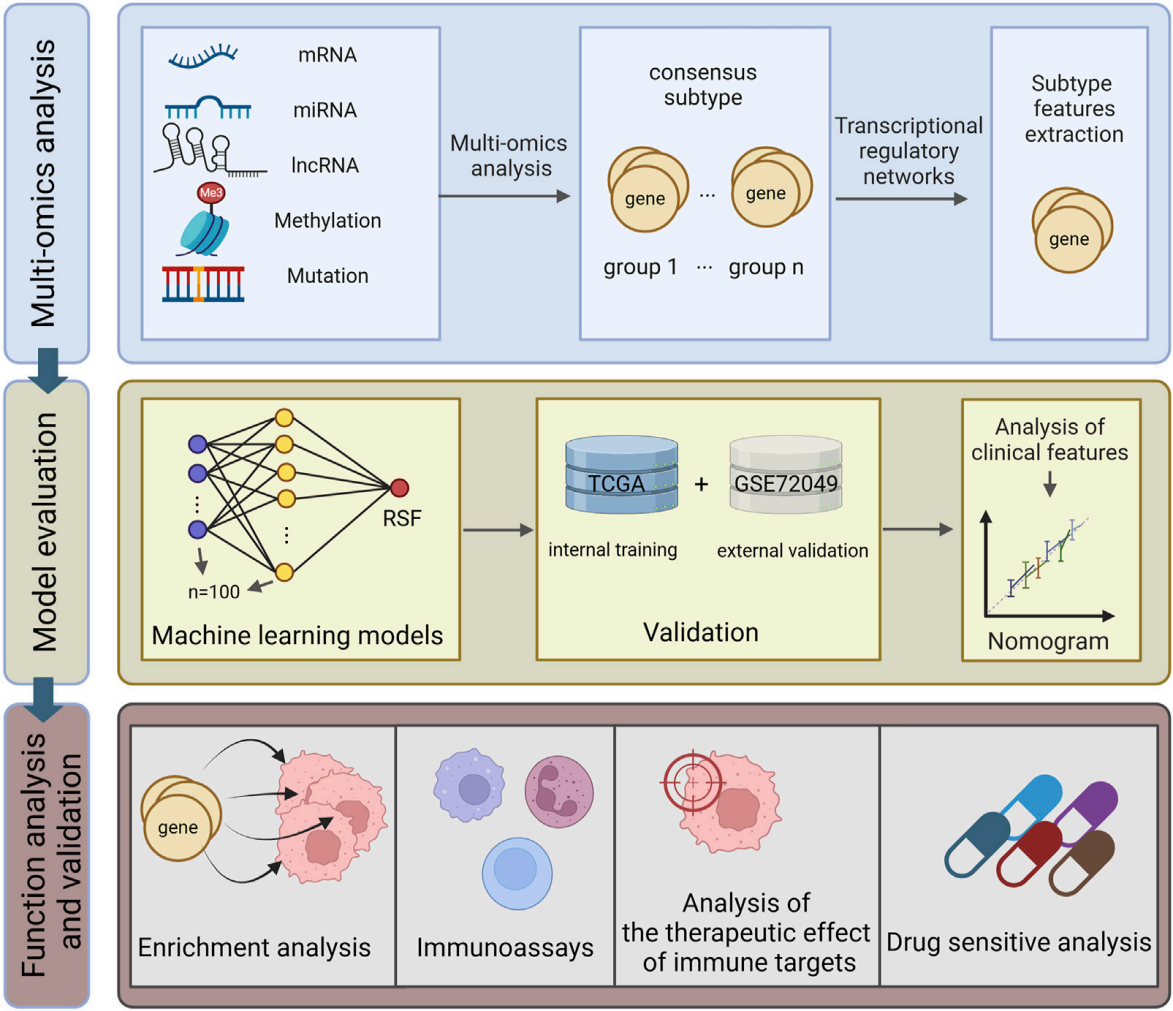
Research flowchart.

All analytical processes are illustrated in the flowchart (Figure 1).

### 3.1 Multi-omics integration reveals two distinct molecular subtypes of LUAD

**FIGURE 2:**
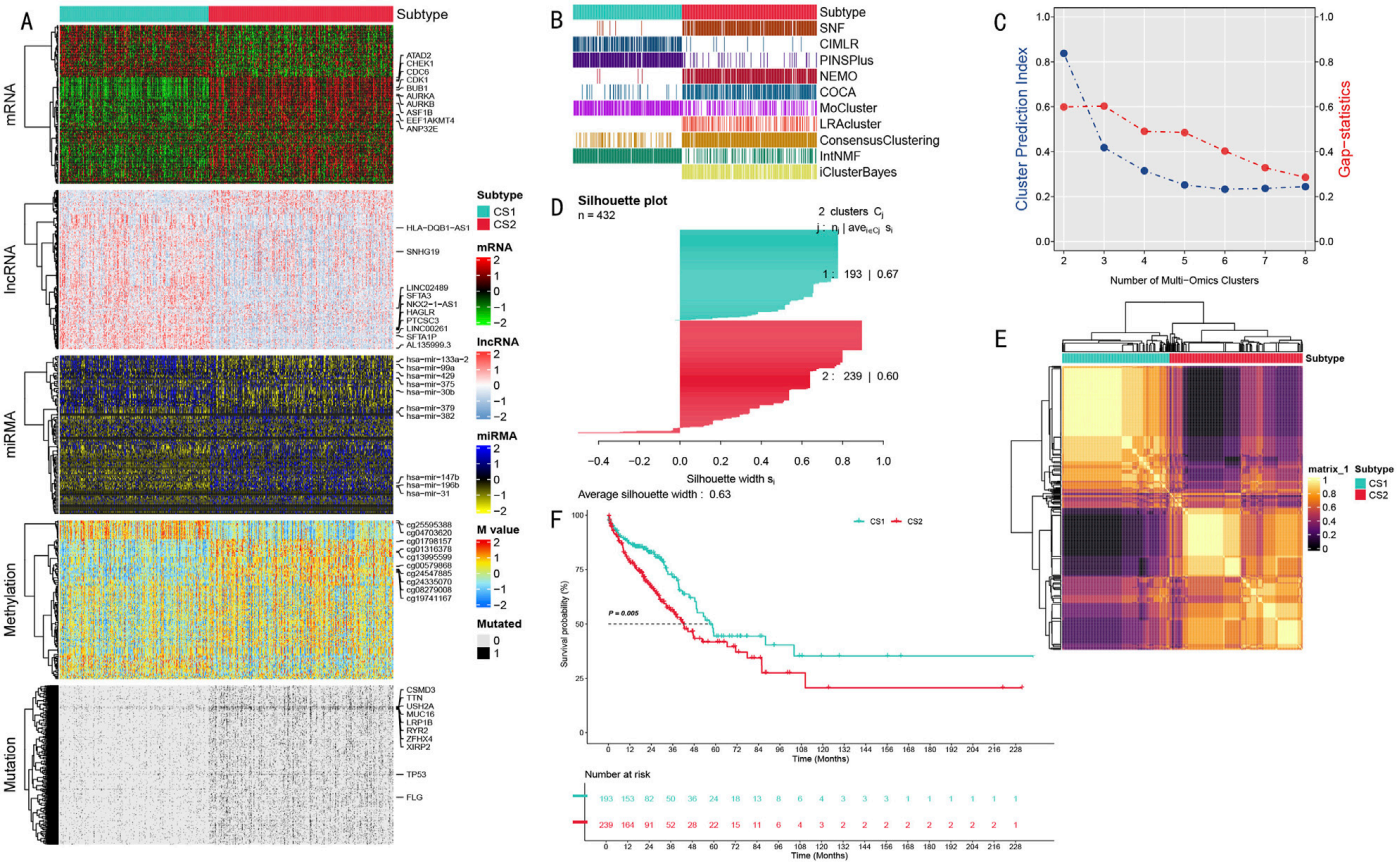
Multi-omics Integration Analysis Results of LUAD Molecular Subtypes. **(A)** Multi-omics feature heatmap showing characteristic differences between CS1 and CS2 subtypes in mRNA, long non-coding RNA, miRNA expression, DNA methylation, and somatic mutations; **(B)** Comparison of subtyping results from different multi-omics integration methods, showing 10 clustering algorithms and their integration results; **(C)** Determination of optimal cluster number based on Gap statistics and clustering prediction indices; **(D)** Silhouette analysis validating the robustness of the two-class scheme, with an average silhouette width of 0.63; **(E)** Molecular feature correlation heatmap showing sample similarity within subtypes and differences between subtypes; **(F)** Kaplan-Meier survival analysis showing significant prognostic differences between CS1 and CS2 subtypes (P = 0.005) with follow-up extending to 228 months.

To comprehensively characterize the molecular heterogeneity of LUAD, we implemented a systematic multi-omics integration analysis strategy. We initially selected 1,500 features with the highest variation from each omics data level (for mutation data, we selected the 1,500 sites with the highest mutation frequency). By applying ten established clustering algorithms to LUAD samples (Figure 2B), we established a robust consensus subtyping (CS) scheme. Through systematic evaluation of clustering schemes from k = 2 to k = 8 using Gap statistics and clustering prediction indices, both metrics achieved optimal values at k = 2, providing strong statistical support for a two-subtype classification scheme (Figure 2C).

The multi-omics feature landscape (Figure 2A) clearly demonstrated significant molecular pattern differences between these two subtypes. We validated the classification scheme’s robustness through multiple methods, including comparative analysis of different clustering methods (Figure 2B), correlation heatmap analysis (Figure 2E), and silhouette analysis (average silhouette width of 0.63, Figure 2D). This comprehensive analysis ultimately divided the patient population into two subtypes: CS1 (n = 193) and CS2 (n = 239).

These two subtypes exhibited significant molecular characteristic differences across all data types (Figure 2A), including mRNA expression level differences in cell cycle regulatory genes (AURKA, AURKB, BUB1, and CDK1), expression profile differences in long non-coding RNAs (LINC00261 and SFTA1P), expression differences in microRNAs (particularly hsa-mir-31 and hsa-mir-196b), differences in DNA methylation patterns, and mutation frequency differences in cancer-associated genes (especially TP53 and MUC16). Most importantly, these molecular-level differences were closely associated with clinical prognosis. Survival analysis revealed significantly different prognostic patterns between the two subtypes (P = 0.005), with the CS1 subtype consistently showing poorer survival outcomes throughout the 228-month follow-up period (Figure 2F).

### 3.2 Biological characteristics and immune microenvironment analysis of different subtypes

**FIGURE 3:**
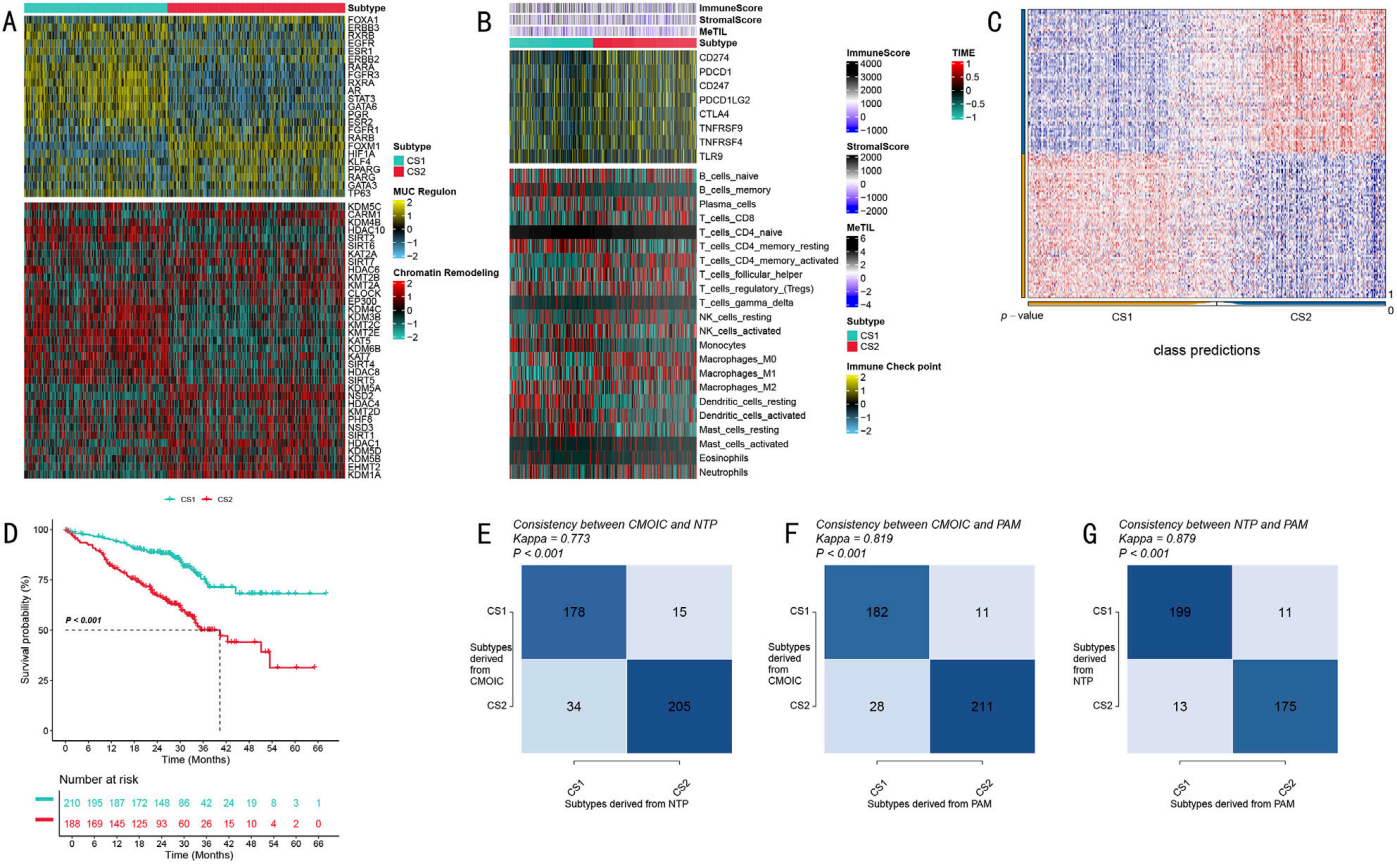
Biological Characteristics and Validation Analysis of LUAD Molecular Subtypes. **(A)** Expression profile heatmap of MUC family and chromatin remodeling-related genes, with upper portion showing MUC regulatory region gene expression and lower portion showing chromatin remodeling-related gene expression patterns; **(B)** Tumor immune microenvironment characteristic analysis heatmap, displaying immune scores, stromal scores, and immune cell infiltration components from top to bottom, with color scale indicating relative abundance; **(C)** Classification prediction matrix showing prediction probability distribution of sample classification; **(D)** Validation cohort Kaplan-Meier survival analysis showing survival differences between the two subtypes, including risk number table; **(E–G)** Classification method consistency validation, showing cross-validation results and Kappa consistency coefficients for CMOIC, NTP, and PAM methods.

Through systematic functional annotation analysis, we revealed significant biological characteristic differences between the two LUAD molecular subtypes. Transcriptional regulatory network analysis demonstrated distinct expression regulatory patterns between the two subtypes, centered on MUC family genes and chromatin remodeling-related genes (Figure 3A). The heatmap in Figure 3A vividly illustrates the expression profile differences of MUC regulatory region genes and chromatin remodeling-related genes, with the upper portion showing MUC family gene expression patterns and the lower portion displaying differential expression characteristics of chromatin remodeling-related genes.

To deeply analyze tumor immune microenvironment characteristics, we conducted comprehensive quantitative analysis using multiple algorithms. Through the integration of ESTIMATE algorithm scores, MeTIL index, and CIBERSORT cell component analysis results, we discovered unique immune cell infiltration characteristics in both subtypes (Figure 3B). Figure 3B presents these differences in heatmap form, displaying from top to bottom the differential distribution of immune scores, stromal scores, and various immune cell infiltration components, with color intensity reflecting relative abundance levels.

To ensure the reliability of our subtyping results, we implemented a rigorous cross-validation strategy. The classification prediction matrix (Figure 3C) demonstrates the prediction probability distribution of sample classification, validating the accuracy of our typing. Survival analysis in the independent validation cohort showed that CS1 subtype patients exhibited significantly better survival benefits compared to CS2 subtype patients (P < 0.001, Figure 3D). The Kaplan-Meier survival curves clearly demonstrate the survival differences between the two subtypes, accompanied by detailed risk number tables.

Notably, through cross-validation using three independent classification methods - CMOIC, NTP, and PAM - we obtained highly consistent classification results (Kappa values of 0.773, 0.819, and 0.879, respectively; Figures 3E–G). The high consistency among these three methods strongly supports the robustness and reliability of this molecular subtyping system. Figures 3E–G detail the cross-validation results of these three classification methods, including their respective Kappa consistency coefficients, further confirming the accuracy of the typing system.

### 3.3 Construction and performance evaluation of random survival forest-based prognostic model

**FIGURE 4:**
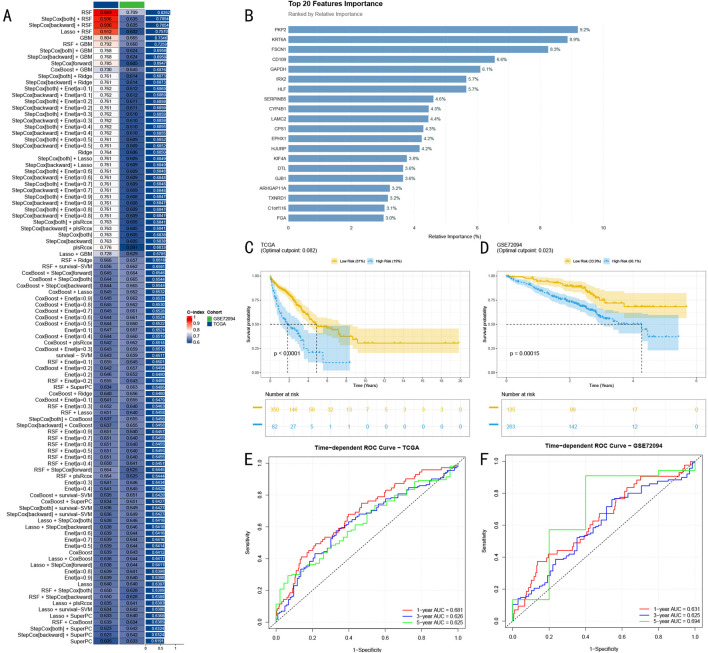
Construction and Validation of Random Survival Forest Prognostic Model. **(A)** Machine learning model performance heatmap showing predictive efficacy of 100 models across different validation sets, with color intensity indicating C-index (0–1); **(B)** Top 20 predictive features selected by RSF model, with bar chart showing relative importance of key genes, blue indicating high importance (>5%), red indicating moderate importance; **(C)** Kaplan-Meier survival analysis in TCGA-LUAD training set (n = 432), showing survival differences between high and low-risk groups; **(D)** Survival analysis results in GSE72094 validation set (n = 398); **(E)** Time-dependent ROC curves in TCGA-LUAD training set (AUC: 0.681, 0.626, and 0.625); **(F)** ROC curves in GSE72094 validation set (AUC: 0.631, 0.625, and 0.694).

We conducted a systematic performance evaluation of 100 machine learning model combinations, visualizing the predictive efficacy of different models across validation sets through a heatmap (Figure 4A). Each row in the heatmap represents an algorithm combination, each column corresponds to a validation dataset, and color intensity reflects the C-index magnitude (0–1). Comprehensive comparison revealed that the RSF model demonstrated optimal predictive performance.

Through variable importance analysis of the RSF model, we successfully identified 20 features with high predictive value (Figure 4B). Among these, seven genes including PKP2, KRT6A, and FSCN1 showed significantly higher relative importance exceeding 5%, marked in blue in the bar chart, while other moderately important features are shown in red, with all features arranged in descending order of importance.

In the TCGA-LUAD training cohort (n = 432), RSF model-based risk scores stratified patients into high and low-risk groups. Kaplan-Meier survival analysis revealed significant survival differences between the groups (P < 0.0001, Figure 4C). Time-dependent ROC curve analysis demonstrated excellent accuracy in 1-year, 3-year, and 5-year survival predictions, achieving AUC values of 0.681, 0.626, and 0.625 respectively (Figure 4E).

To rigorously assess the model’s generalization capability, we conducted validation in the independent GSE72094 cohort (n = 398). Results demonstrated sustained significant predictive power (P = 0.00015, Figure 4D), with stable performance across different time points, showing AUC values of 0.631, 0.625, and 0.694 for 1-year, 3-year, and 5-year predictions respectively (Figure 4F). These results strongly confirm the stable predictive efficacy and promising clinical application potential of our developed RSF model.

### 3.4 Multi-dimensional validation analysis of RSF prognostic model

**FIGURE 5:**
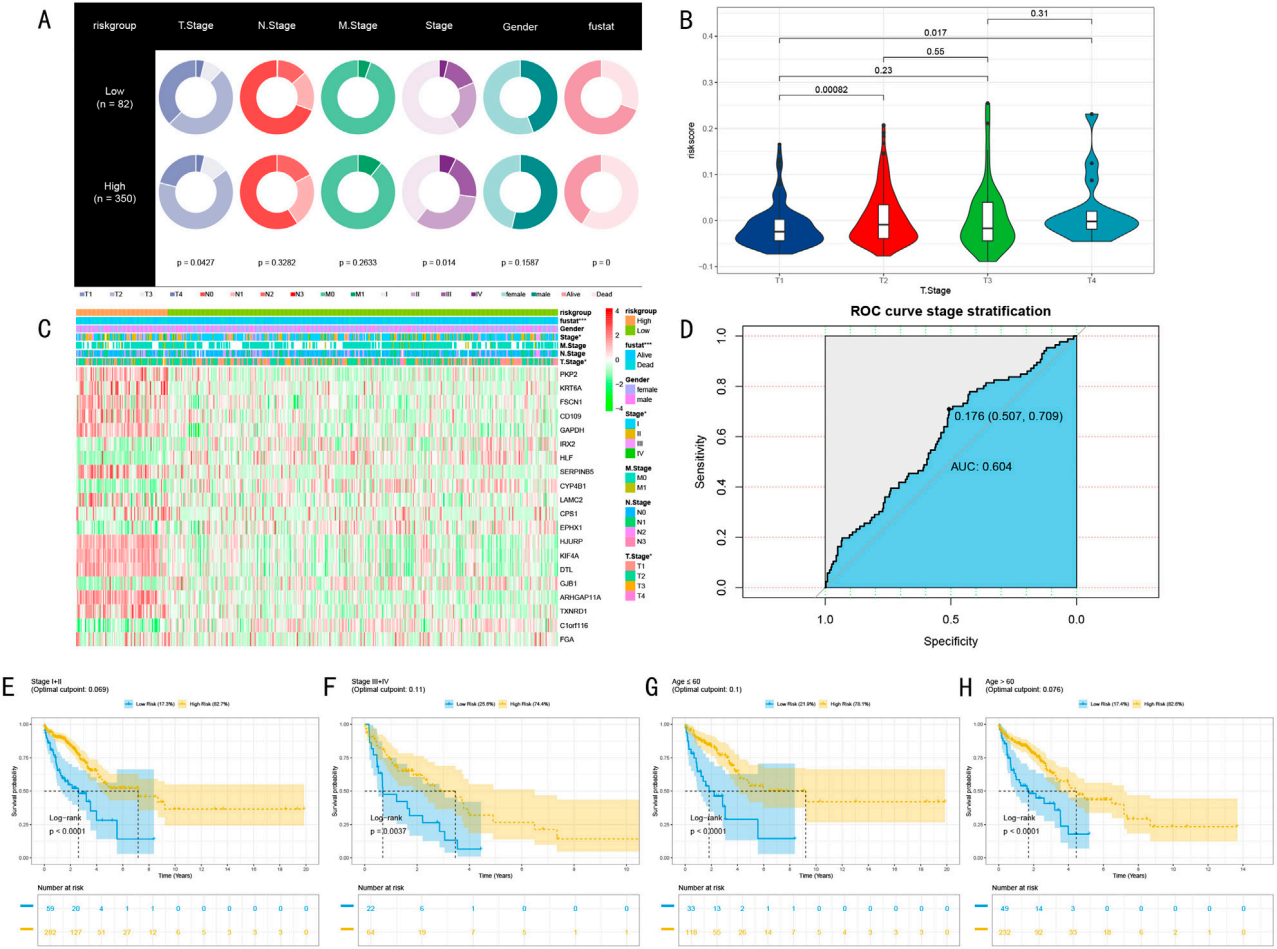
Multi-dimensional Validation Analysis of RSF Prognostic Model. **(A)** Pie charts showing distribution differences of clinical characteristics between high and low-risk groups, displaying differences in TNM staging, clinical staging, gender, and Fustat indicators; **(B)** Chi-square analysis of risk score distribution differences across T stages; **(C)** Expression heatmap of 20 marker genes across different risk groups and clinical phenotypes, showing from top to bottom: Fustat indicators, gender, clinical staging, M staging, N staging, T staging (*P < 0.05, **P < 0.01, ***P < 0.001); **(D)** ROC curve analysis of stratification capability between early and late-stage patients; **(E–H)** Kaplan-Meier survival analysis of clinical stage subgroups and age subgroups, including risk number tables and log-rank test P-values.

We systematically evaluated the clinical utility of the RSF risk prediction model through multi-layered validation analyses. Initially, we compared the distribution of clinical characteristics between high and low-risk groups (High: n = 350, Low: n = 82) (Figure 5A). Pie chart analysis revealed significant differences between the groups in T stage, clinical stage, and Fustat indicators (p < 0.05, p < 0.05, p < 0.001, respectively).

Further analysis of risk score distribution across different T stages revealed significant differences between T1 stage and both T2 and T4 stage patients (P < 0.05, Figure 5B). This finding particularly highlighted the clinical predictive value of risk scores in early-stage (T1) patients. Our constructed marker gene expression heatmap clearly demonstrated the expression patterns of these genes across different risk groups and clinical phenotypes (Figure 5C). The heatmap revealed significant expression differences in clinical staging and T staging (P < 0.05), with even more pronounced differences in Fustat indicators (P < 0.001).

To assess the model’s ability to predict disease progression, we employed ROC curve analysis to evaluate the risk score’s stratification efficacy between early and late-stage patients (Stage I + II vs. III + IV) (Figure 5D). Results demonstrated good stratification capability (AUC = 0.604, 95% CI: 0.507–0.709, criterion = 0.176). More importantly, survival analysis in clinical stage subgroups and age subgroups showed significant predictive value across early-stage (I + II), late-stage (III + IV), non-elderly (age≤60), and elderly (age>60) groups (p < 0.005, Figures 5E–H). These multi-dimensional validation results strongly support the clinical application potential of the RSF risk prediction model.

### 3.5 Independent prognostic value assessment and nomogram construction

**FIGURE 6:**
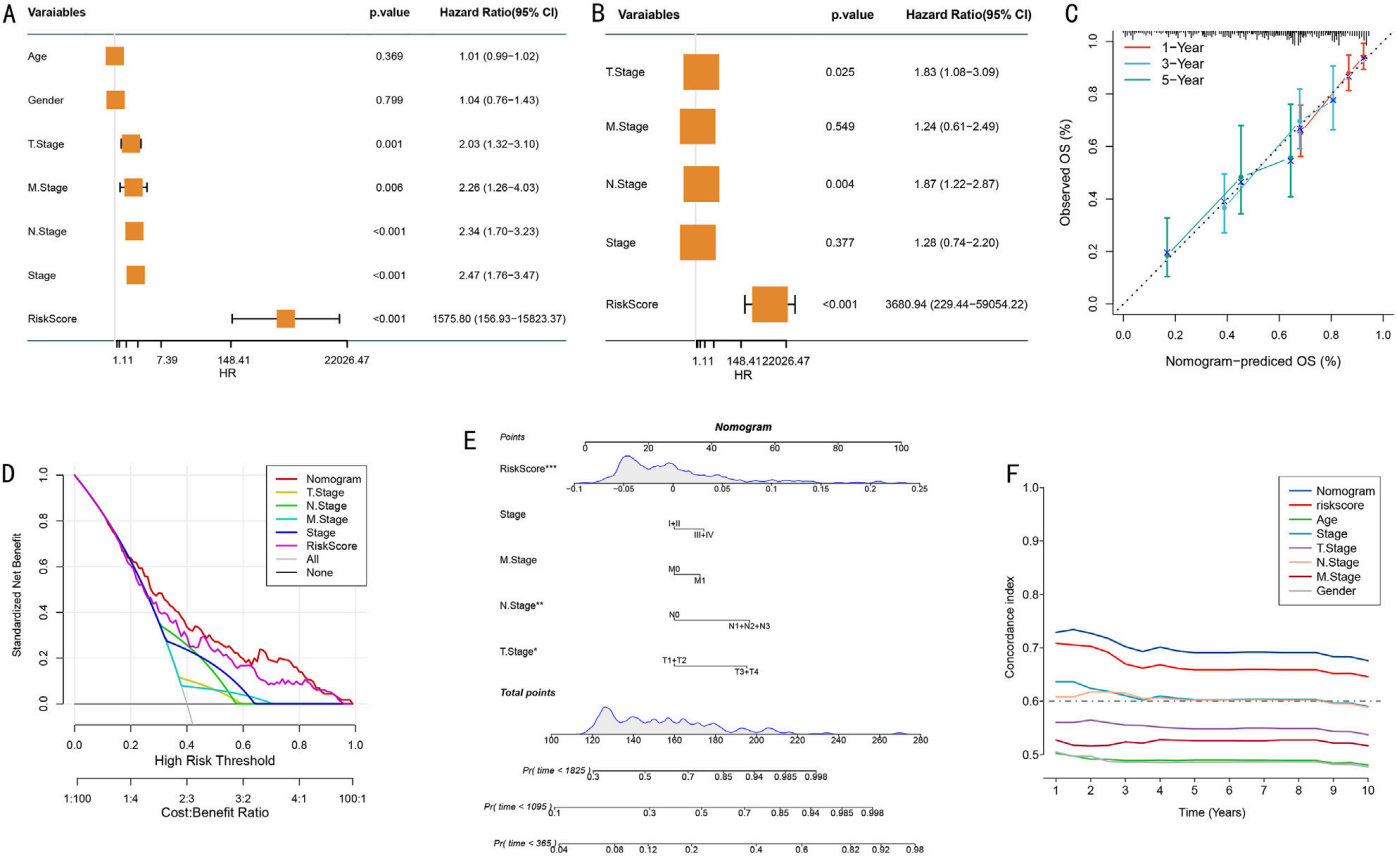
Construction and Evaluation of Integrated Prognostic Model. **(A)** Forest plot of univariate Cox regression analysis showing hazard ratios and 95% confidence intervals for various clinical characteristics; **(B)** Forest plot of multivariate Cox regression analysis confirming independent prognostic factors; **(C)** Calibration curves for nomogram model’s 1-year, 3-year, and 5-year survival probability predictions; **(D)** Decision curve analysis (DCA) of different prediction strategies; **(E)** Prognostic prediction nomogram integrating TNM staging, clinical scores, and risk scores (*P < 0.05, **P < 0.01, ***P < 0.001); **(F)** Dynamic comparison of time-dependent C-indices between nomogram model and single prognostic factors.

To systematically evaluate the independent prognostic value of the risk prediction model, we first conducted comprehensive Cox proportional hazards regression analysis. Univariate analysis results, presented as a forest plot (Figure 6A), revealed TNM staging, clinical staging, and risk scores as significant prognostic factors (all P < 0.01), with T stage showing a relatively lower hazard ratio. Multivariate Cox regression analysis further confirmed the independent prognostic value of T stage, N stage, and risk score (Figure 6B).

Based on the confirmed independent prognostic factors, we constructed an integrated nomogram prediction model. Calibration curve analysis evaluated the model’s prediction accuracy, demonstrating excellent calibration in 1-year (red), 3-year (blue), and 5-year (green) survival predictions (Figure 6C). Decision curve analysis (DCA) further confirmed that the integrated nomogram model provided greater net benefit for clinical decision-making compared to single prognostic factors (Figure 6D).

We established a comprehensive visualization nomogram incorporating all independent prognostic factors (Figure 6E), where risk score, N stage, and T stage again demonstrated significant independent prognostic value (P < 0.001, P < 0.01, P < 0.05, respectively). Dynamic analysis of time-dependent C-index showed that the nomogram model’s prediction accuracy (C-index>0.65) consistently outperformed single prognostic factors throughout the follow-up period (Figure 6F). This integrated prognostic prediction tool provides clinicians with an intuitive, accurate individualized prognostic assessment approach.

### 3.6 Functional annotation and pathway enrichment analysis reveal molecular biological mechanisms

**FIGURE 7:**
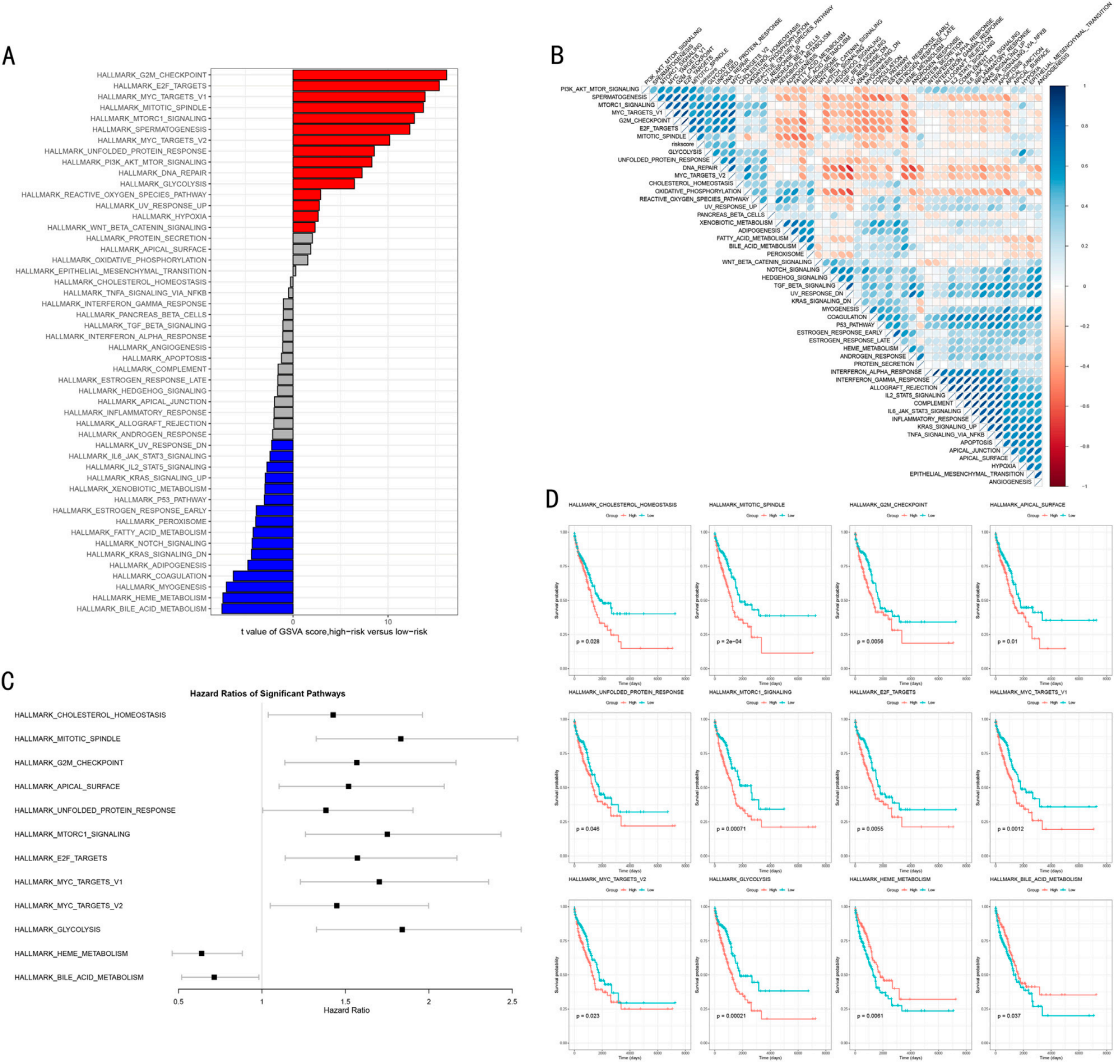
Molecular Mechanism Functional Analysis of RSF Model. **(A)** GSVA differential pathway waterfall plot showing significantly different biological pathways between high and low-risk groups, with red and blue indicating upregulated pathways in high-risk and low-risk groups respectively; **(B)** Correlation heatmap between risk scores and pathway activities, with red and blue indicating positive and negative correlations; **(C)** Forest plot of key pathway hazard ratios showing hazard ratios and 95% confidence intervals for each pathway; **(D)** Kaplan-Meier survival analysis of 12 important pathways, including risk number tables and log-rank test P-values.

Based on the risk stratification results from the RSF model, we conducted systematic functional enrichment analysis to elucidate its molecular biological foundations. Gene Set Variation Analysis (GSVA) revealed risk stratification-specific signaling pathway activity characteristics (Figure 7A). The waterfall plot clearly demonstrates significantly different biological pathways between high and low-risk groups, where the high-risk group significantly activated 15 signature pathways (FDR<0.05), primarily including cell cycle regulation (G2M CHECKPOINT) and MYC targets (MYC TARGETS_V1, MYC TARGETS_V2) related pathways. In contrast, the low-risk group characteristically activated 16 pathways, including P53 pathway, IL-6/JAK/STAT3 signaling pathway, Notch signaling pathway, and KRAS pathway.

Through correlation analysis between risk scores and pathway activities, we constructed a comprehensive functional regulatory network landscape (Figure 7B). The red and blue colors in the heatmap represent positive and negative correlations respectively, with color intensity reflecting correlation strength, further validating our findings.

To evaluate the clinical prognostic significance of key pathways, we focused on analyzing 12 most significant signaling pathways, encompassing metabolism-related (GLYCOLYSIS, HEME METABOLISM, BILE ACID METABOLISM), cell cycle and division-related (G2M CHECKPOINT, MITOTIC_SPINDLE), gene expression and transcriptional regulation-related (E2F targets, MYC targets) and other critical pathways. Hazard ratio (HR) analysis (Figure 7C) confirmed that heme metabolism (HEME METABOLISM) and bile acid metabolism (BILE ACID METABOLISM) are important adverse prognostic factors (HR > 1). Kaplan-Meier survival analysis (Figure 7D) further validated that high activity in these two pathways is significantly associated with poorer overall survival (P < 0.05), while other pathways demonstrated protective prognostic effects.

### 3.7 Analysis of immune microenvironment characteristics and their association with risk scores

**FIGURE 8:**
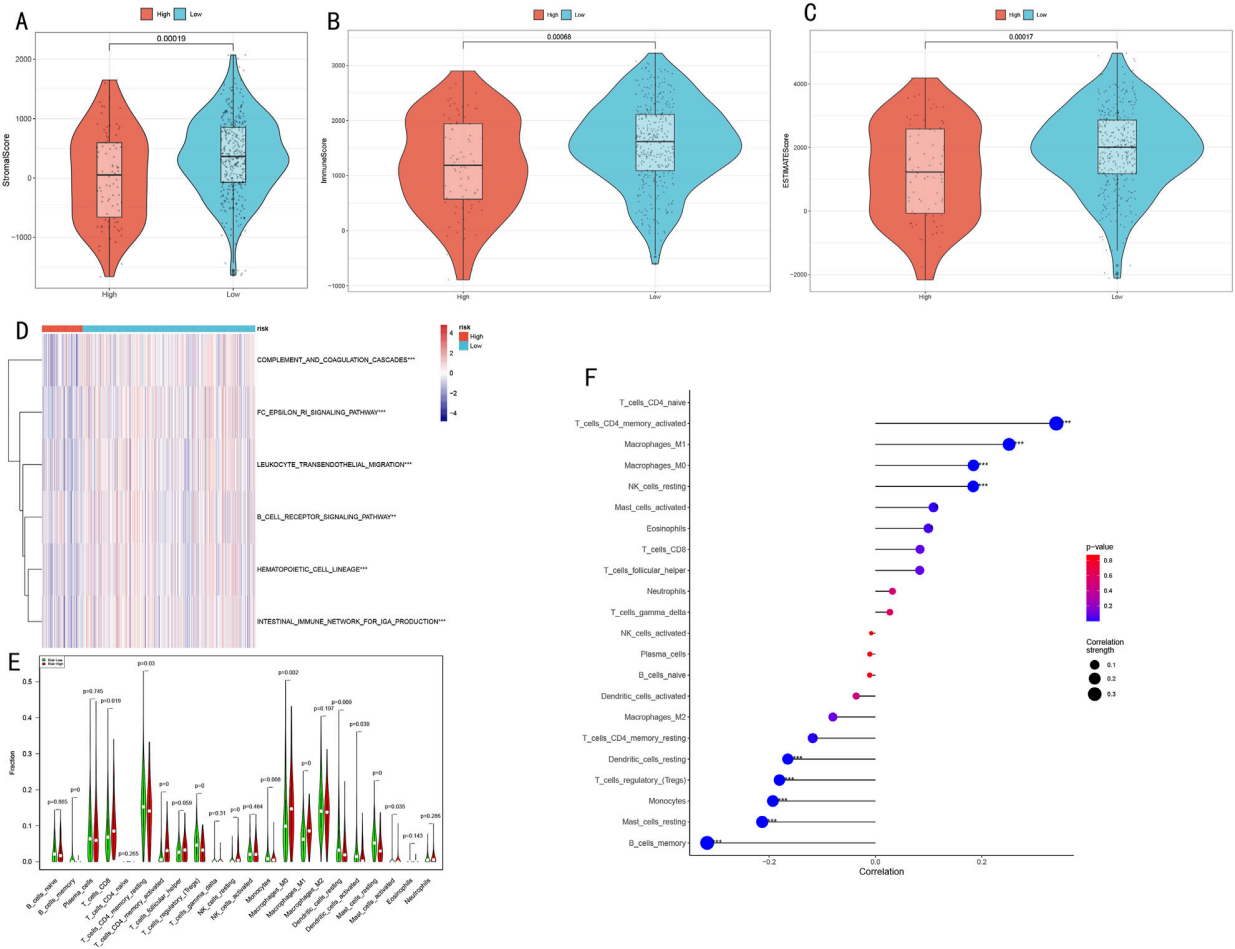
Analysis of Immune Microenvironment Characteristics and Their Association with Risk Scores. **(A–C)** Immune microenvironment differences between high and low-risk groups assessed by ESTIMATE algorithm, including stromal score, immune score, and overall score; **(D)** Activity heatmap of differential immune-related pathways (*P < 0.05, **P < 0.01, ***P < 0.001); **(E)** Violin plots showing infiltration proportion differences of 22 immune cell types (*P < 0.05, **P < 0.01, ***P < 0.001); **(F)** Correlation plot between risk scores and immune cell content, where dot size represents absolute correlation coefficient and color indicates correlation direction and significance.

Our multi-dimensional analysis thoroughly explored the relationship between risk scores and tumor immune microenvironment. Initially, ESTIMATE algorithm assessment results (Figures 8A–C) demonstrated that the low-risk group exhibited significantly higher stromal scores, immune scores, and overall scores compared to the high-risk group (p < 0.001), indicating more active immune responses and richer stromal components in the low-risk group.

ssGSEA algorithm analysis identified six immune-related pathways with significant differences between high and low-risk groups (Figure 8D), including immune response and inflammation-related pathways (complement and coagulation cascades, FC epsilon RI signaling pathway, leukocyte transendothelial migration), B cell receptor signaling pathway, hematopoietic cell lineage, and intestinal IgA production immune network. The heatmap clearly illustrates the activity differences of these pathways across risk groups.

CIBERSORT algorithm analysis of immune cell infiltration characteristics (Figure 8E) revealed three major differences:

1. Memory B cells, regulatory T cells, M1 macrophages, and resting mast cells were significantly decreased in the high-risk group (P < 0.001);
2. Activated memory CD4^+^ T cells and resting NK cells were more abundant in the high-risk group (P < 0.001);
3. The low-risk group was enriched with monocytes, M0 macrophages, activated dendritic cells, CD8^+^ T cells, resting memory CD4^+^ T cells, resting dendritic cells, and activated mast cells (P < 0.05).

Correlation analysis between risk scores and immune cell content (Figure 8F) revealed:

- Significant positive correlations with memory CD4^+^ T cells, CD4^+^ T cells, M1/M0 macrophages, and NK cells (P < 0.001).
- Significant negative correlations with dendritic cells, T cells, monocytes, mast cells, and B cells (P < 0.001).

These results suggest potential immune suppression or dysregulation in the high-risk group, while the low-risk group may possess more effective immune regulatory mechanisms. The risk score serves as an effective indicator for quantifying LUAD patients’ immune status, reflecting significant immunological landscape differences between patients with different risk levels.

### 3.8 Immunotherapy response prediction and immune function assessment

**FIGURE 9:**
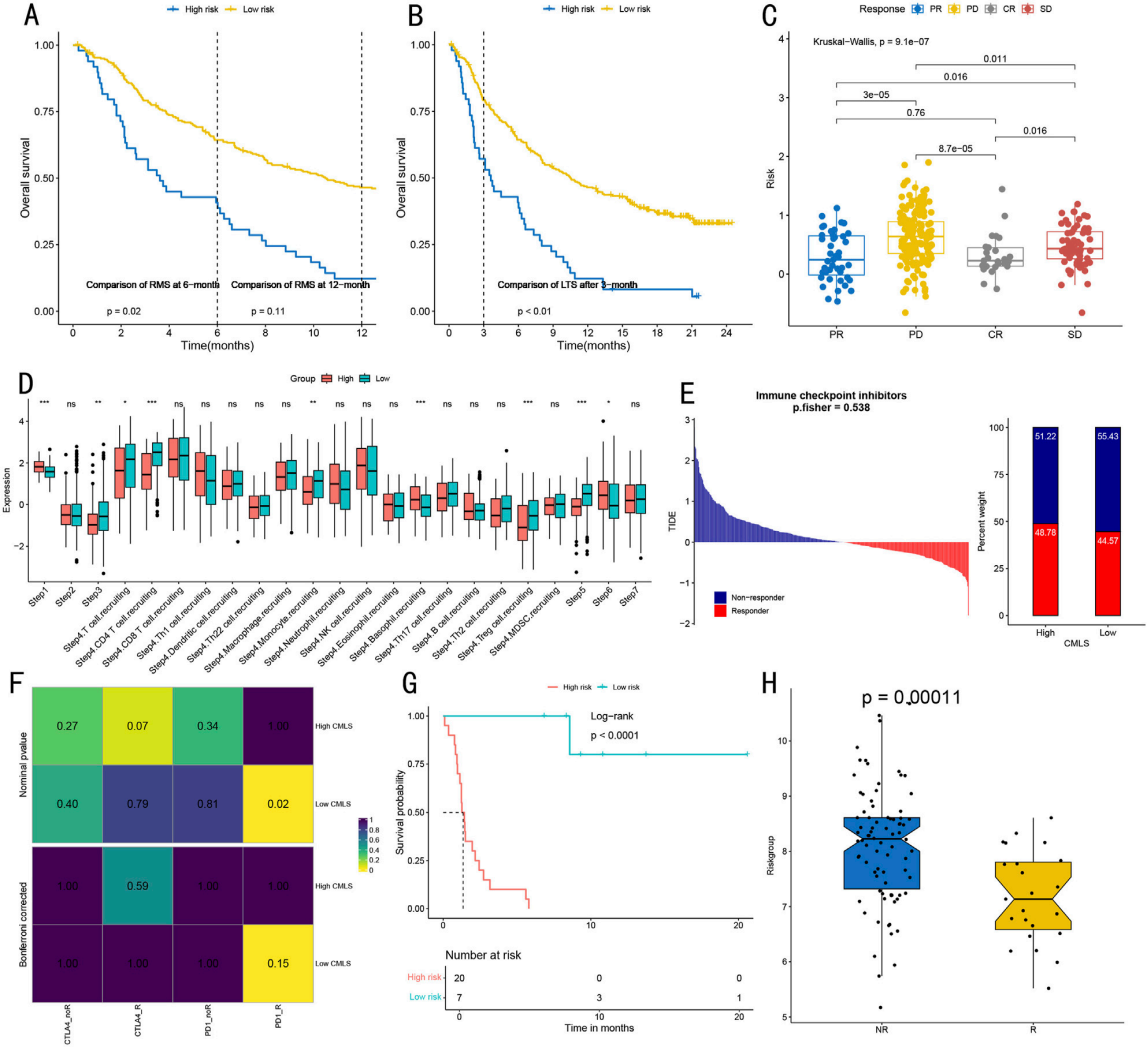
Immunotherapy Response Prediction and Immune Function Assessment. **(A)** Survival analysis of high and low-risk groups within restricted survival times, showing survival curves and log-rank test P-values at 6 and 12 months (p = 0.02, p = 0.11). **(B)** Long-term survival analysis (24 months) after 3 months for high and low-risk groups, showing significant differences (p < 0.01). **(C)** Kruskal–Wallis test evaluating associations between different treatment response groups (CR complete response/PR partial response/SD stable disease/PD progressive disease) and risk scores, showing statistical significance of intergroup differences. **(D)** Quantitative analysis of tumor-infiltrating immune cell levels in seven steps of the cancer immunity cycle, including detailed visualization of 17 immune cell subgroups in step four. Statistical significance: *P < 0.05, **P < 0.01, ***P < 0.001. **(E)** TIDE algorithm prediction of immune checkpoint inhibitor treatment response in High-CMLS and Low-CMLS groups, showing distribution of Responders and Non-responders (p = 0.538). **(F)** Correlation heatmap from SubMap algorithm analysis, showing association strength between high/low CMLS groups and different immunotherapy response types, with values and color intensity representing correlation degree. **(G)** Survival analysis based on CMLS grouping, showing survival differences between high and low-risk groups (Log-rank p < 0.0001), including risk number table for 20-month follow-up period. **(H)** Box plot comparison of scores between immunotherapy response (R) and non-response (NR) groups, showing significant differences (p = 0.00011), with each point representing one sample.

To validate the predictive value of the RSF model for immunotherapy response, we conducted systematic verification across multiple independent cohorts. Initial assessment of the association between risk scores and treatment response (Figures 9A, B) demonstrated that the high-risk group exhibited lower overall survival than the low-risk group within both 6-month and 12-month restricted mean survival times. This difference was particularly significant for long-term survival beyond 3 months (P < 0.01). The analysis of treatment response (CR/PR/SD/PD) differences in risk scores (Figure 9C) revealed significant variations between PD and both PR and CR groups (P < 0.05), indicating excellent predictive capability for disease progression or remission.

To evaluate immune function characteristics, we quantitatively visualized immune cell infiltration levels across different cancer cycle stages (Figure 9D). Steps one, three, five, and six demonstrated significant immune cell infiltration levels (all P < 0.05). Additionally, step four showed high infiltration levels of T cells, CD4^+^ T cells, monocytes, basophils, and regulatory T cells (all P < 0.05). Immune checkpoint inhibitor treatment response prediction (Figure 9E) revealed that the proportion of ICB responders in the High-CMLS group approximated that of the Low-CMLS group. SubMap algorithm analysis indicated strong correlation between the Low-CMLS group and PD-1 inhibitor treatment response (Figure 9F).

Survival analysis further validated the prognostic value of CMLS-based grouping, with the low-risk group demonstrating significantly better survival benefits (p < 0.0001, Figure 9G). In the independent validation cohort, the immunotherapy response group showed significantly lower scores than the non-response group (p = 0.00011, Figure 9H), further supporting the model’s value in predicting immunotherapy response.

### 3.9 Drug sensitivity analysis

**FIGURE 10:**
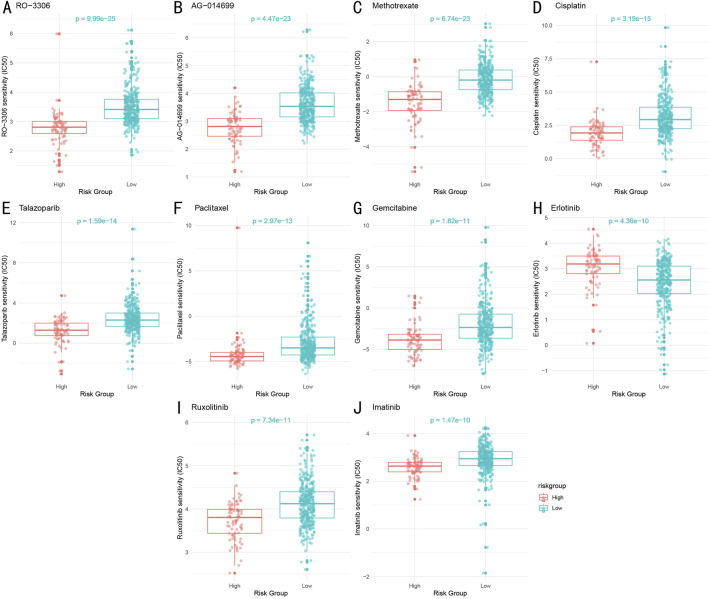
Drug Sensitivity Analysis Between High and Low-Risk Groups **(A–J)**. Sensitivity comparison of ten key therapeutic drugs between high and low-risk groups. Box plots show distribution of predicted IC50 values, with lower IC50 values indicating higher drug sensitivity. Statistical significance determined by Wilcoxon rank-sum test.

Through systematic IC50 value prediction, we identified ten potential therapeutic drugs showing significant sensitivity differences between high and low-risk groups (Figures 10A–J). These drugs can be classified into four categories:

1. Chemotherapy drugs: Methotrexate (P = 6.74e-23), Cisplatin (P = 3.19e-15), Paclitaxel (P = 2.97e-13), and Gemcitabine (P = 1.82e-11).
2. Targeted therapy drugs: Erlotinib (P = 4.36e-10), Ruxolitinib (P = 7.34e-11), and Imatinib (P = 1.47e-10).
3. PARP inhibitors: AG-014699 (P = 4.47e-23) and Talazoparib (P = 1.59e-14).
4. CDK inhibitor: RO-3306 (P = 9.99e-25).

Notably, except for the targeted therapy drug Erlotinib, the low-risk group demonstrated higher sensitivity to most drugs compared to the high-risk group. Both groups showed significant sensitivity to the chemotherapy drug Paclitaxel.

## 4 Discussion

### 4.1 Primary research findings

Through multi-omics integrated analysis, this study identified two distinct molecular subtypes of LUAD (CS1 and CS2). These subtypes exhibited significant differences across multiple omics data, including gene expression, DNA methylation, miRNA, and lncRNA, with CS2 subtype patients demonstrating superior immune activity and longer survival duration (P = 0.005). The subtyping results showed robustness across various clustering methods (average silhouette width 0.63) and received consistent validation in independent cohorts. Based on these subtyping results, we developed multiple machine learning-based prognostic models to further quantify patient risk and guide clinical decision-making. Among these, the RSF model demonstrated excellent predictive performance in both the training set (TCGA-LUAD) and validation set (GSE72094), with time-dependent ROC curve AUC values of 0.681 vs. 0.631 (1-year), 0.626 vs. 0.625 (3-year), and 0.625 vs. 0.694 (5-year), respectively. The model identified 20 critical feature genes, with PKP2, KRT6A, and FSCN1 showing the highest contribution. These genes exhibited significantly different expression patterns between high and low-risk groups, suggesting their potential crucial roles in LUAD development and progression. Further analysis revealed that risk scores significantly influenced patients’ immune microenvironment characteristics.

Immune microenvironment analysis demonstrated immunosuppressive states in the high-risk group, with significantly reduced infiltration of CD8^+^ T cells, M1 macrophages, and dendritic cells (P < 0.05), while the low-risk group exhibited more active immune responses. Risk scores showed significant correlations with immune scores, stromal scores, and immune cell infiltration levels (P < 0.001). These findings, along with subsequent immunotherapy response prediction results, suggest that low-risk group patients may be more suitable for PD-1 inhibitor treatment (Shiravand et al., 2022). Finally, to further explore the clinical application value of risk scores, we conducted drug sensitivity analysis. Results revealed that the low-risk group showed higher sensitivity to chemotherapy and targeted drugs including Cisplatin, Paclitaxel, and Erlotinib (P < 0.01), while the high-risk group may require alternative treatment strategies. These results indicate significant differences in drug response between different risk groups, providing important evidence for developing personalized treatment plans.

### 4.2 Biological significance of research findings

#### 4.2.1 Critical role of epigenetic regulation in LUAD molecular subtyping

The key genes identified through our RSF model (PKP2, KRT6A, FSCN1, etc.) play crucial roles in LUAD development and progression. PKP2 (plakophilin 2), a member of the plakophilin family, is subject to dual regulation by DNA methylation and histone modifications (Niell et al., 2018). Our analysis revealed decreased PKP2 expression in the high-risk group, potentially associated with elevated methylation levels in its promoter region. This downregulation of PKP2 disrupts intercellular connections and promotes tumor cell invasion and metastasis, consistent with previous studies identifying PKP2 as a tumor suppressor (Cheng C. et al., 2021).

KRT6A (keratin 6A) and FSCN1 (fascin actin-bundling protein 1) expression regulation involves complex epigenetic networks (Chen et al., 2022; Chang et al., 2023). Our study found abnormally high expression of these genes in the high-risk group, significantly correlating with poor prognosis. Further analysis suggested that this upregulation might be related to enhanced activity of the histone demethylase KDM5B, which promotes transcriptional activation by removing the repressive H3K4me3 mark. This finding reveals the regulatory mechanism of epigenetic modifications in LUAD progression.

Notably, we observed that epigenetic modification patterns closely correlate with tumor heterogeneity. Different molecular subtypes exhibited unique DNA methylation profiles and histone modification characteristics, suggesting that this epigenetic heterogeneity might be a key factor in treatment response variations (Sadida et al., 2024). For instance, CS1 subtype patients generally exhibited genome-wide hypomethylation (Wang X. et al., 2021), potentially explaining their poorer prognosis through the abnormal activation of oncogenes.

#### 4.2.2 Association between immune microenvironment characteristics and clinical prognosis

Our study revealed significant characteristics of the LUAD immune microenvironment and their clinical implications. Regarding immune cell infiltration patterns, the low-risk group demonstrated higher levels of CD8^+^ T cells, M1 macrophages, and dendritic cells infiltration, with this “hot” tumor microenvironment significantly correlating with better prognosis. In contrast, the immunosuppressive state of the high-risk group (increased regulatory T cells proportion, decreased effector immune cells) might be a crucial factor in their poor prognosis.

Immune scores showed a significant positive correlation with patient prognosis. The high immune scores in the low-risk group not only reflected more active anti-tumor immune responses but also indicated better treatment responses (Sui et al., 2020). This finding aligns with several recent studies, emphasizing the importance of tumor immune state assessment in prognostic evaluation.

Particularly noteworthy is the close correlation between immune microenvironment characteristics and treatment response. Our analysis showed that low-risk group patients with active immune responses demonstrated significantly higher response rates to immune checkpoint inhibitor therapy, providing important guidance for patient selection in immunotherapy while explaining why certain patients respond poorly to immune therapy.

#### 4.2.3 Molecular mechanisms of drug sensitivity differences

The significant drug sensitivity differences between high and low-risk groups likely stem from multiple molecular mechanisms. First, variations in epigenetic states lead to different expression levels of drug targets. For example, the high sensitivity to the EGFR inhibitor Erlotinib in the low-risk group correlates with their EGFR pathway gene expression patterns (Ma et al., 2024). Second, differences in cell cycle regulatory pathway activity influence chemotherapy effectiveness (Sun et al., 2021). We observed higher sensitivity to taxane drugs in the low-risk group, potentially related to their intact G2/M checkpoint pathway.

Key signaling pathway analysis revealed significant activation of MYC and E2F target genes in the high-risk group, potentially leading to cell cycle dysregulation and drug resistance (Gu et al., 2023). Conversely, the integrity of the P53 pathway in the low-risk group helps maintain cell cycle checkpoint functions, increasing chemotherapy sensitivity (Huang and Liu, 2013). Additionally, the activation state of the PI3K/AKT/mTOR pathway influences drug responses (Huang et al., 2019), explaining the varying effectiveness of certain targeted therapies across risk groups.

Based on these findings, we recommend personalizing treatment strategies according to patient risk stratification. For low-risk group patients, conventional chemotherapy combined with immunotherapy may be optimal, while high-risk group patients might require targeted therapy or novel drug combinations. This mechanism-based treatment strategy selection promises to improve therapeutic outcomes and patient prognosis.

### 4.3 Clinical application value

#### 4.3.1 Clinical translation prospects of the prognostic prediction model

The RSF model demonstrated moderate initial predictive performance (C-index: 0.67, AUC: 0.65–0.70), but showed notably improved accuracy in external validation cohorts with longer follow-up periods (5-year AUC: 0.694). In comparison, Yang et al.'s model achieved AUCs of 0.63 and 0.60 for 1-year and 3-year predictions respectively, with a decline to 0.59 for 5-year predictions (Yang et al., 2022). Similarly, Li et al.'s model reported a 5-year AUC of only 0.653 (Li et al., 2022). Compared to these previous models, our approach offers several unique advantages. First, it represents the first integration of epigenetic features in lung cancer prognostic modeling, capturing an additional layer of biological regulation that may influence treatment response. Second, previous models typically lack external validation and immunotherapy response prediction, making their real-world clinical utility uncertain. Our model not only shows improved performance metrics but also reflects the inherent complexity of LUAD biology, prioritizing reproducibility and clinical interpretability over potentially overfitted accuracy metrics.

Our RSF prognostic prediction model demonstrates significant clinical application potential. First, the model integrates molecular characteristics and clinicopathological parameters, showing stable predictive performance in both training and validation sets (AUC>0.6). This predictive accuracy provides clinicians with a reliable decision-support tool. Particularly in early-stage LUAD patients, the model effectively identifies high-risk individuals, providing guidance for adjuvant therapy selection.

The model’s value in treatment plan selection manifests in three aspects: (1) risk scores can predict potential effectiveness of chemotherapy and targeted therapy, aiding optimal treatment strategy selection; (2) molecular subtyping information helps determine immunotherapy suitability; (3) for high-risk patients, the model suggests more aggressive treatment approaches and more frequent follow-up monitoring.

In personalized medicine practice, this model can complement existing clinical guidelines, providing more precise reference for treatment decisions. For instance, risk scores can guide decisions about adjuvant therapy necessity for early-stage (I-II) patients, while helping optimize treatment combinations for advanced patients.

#### 4.3.2 Patient selection strategy for immunotherapy benefits

Based on our findings, we propose a systematic patient selection strategy for immunotherapy. Patients with low risk scores typically possess more active immune microenvironments, characterized by higher CD8^+^ T cell infiltration and lower proportions of immunosuppressive cells, suggesting they are more likely to benefit from immune checkpoint inhibitor therapy. Our prediction model demonstrates superior accuracy in predicting immunotherapy response (AUC>0.7), outperforming traditional methods that rely solely on PD-L1 expression or tumor mutation burden (TMB) (Yarchoan et al., 2019).

The differential immunotherapy response between risk groups appears driven by distinct epigenetic patterns. High-risk tumors showed epigenetic silencing of immune response genes, particularly in antigen presentation and T cell activation pathways. This epigenetic-mediated immunosuppression may create a “cold” tumor microenvironment resistant to PD-1 blockade, suggesting potential benefit from combining epigenetic modifiers with immunotherapy in high-risk patients.

To enhance immunotherapy effectiveness, we recommend: (1) conducting detailed immune microenvironment assessments before treatment, including immune cell composition analysis and immune function scoring; (2) considering initial radiochemotherapy to activate immune responses in patients with lower immune scores before implementing immunotherapy; (3) exploring combined targeted therapy and immunotherapy strategies for patients with specific gene mutations.

### 4.4 Study limitations and future prospects

This study presents several notable limitations. First, although we integrated multiple cohorts from TCGA and GEO databases, the sample size remains relatively limited and primarily represents Western populations, potentially not fully reflecting Asian population characteristics. Second, validation cohorts lack complete multi-omics data, particularly epigenetic modification-related data, limiting comprehensive validation of molecular subtyping results. Regarding technical methods, inherent limitations of computational approaches may affect prediction accuracy, such as potential bias in CIBERSORT algorithm’s immune cell infiltration assessment. Additionally, our drug sensitivity predictions, based primarily on *in vitro* cell line data, may not fully reflect clinical responses due to the absence of tumor microenvironment complexity and patient-specific factors. Future validation through prospective clinical trials will be essential to confirm these computational predictions. Based on these limitations, future research should focus on:

1. Expanding validation cohort size, particularly incorporating more Asian population data
2. Conducting prospective clinical studies to validate prediction model effectiveness
3. Integrating novel omics technologies (e.g., single-cell sequencing, spatial transcriptomics) for deeper tumor heterogeneity analysis
4. Exploring new machine learning algorithms to improve prediction model accuracy
5. Developing early diagnosis and recurrence monitoring research to expand model applications

Additionally, developing standardized testing platforms and clinical decision support systems will facilitate clinical translation. These in-depth studies promise to further improve LUAD patient diagnostic and therapeutic precision, ultimately enhancing patient outcomes.

## 5 Conclusion

Through integrating multi-omics data and advanced machine learning methods, this study successfully constructed an epigenetic feature-based LUAD molecular subtyping system and prognostic prediction model. Our research pioneered the identification of two distinct molecular subtypes (CS1 and CS2) based on epigenetic regulation, confirming their significant differences in immune microenvironment characteristics and clinical prognosis. The RSF prognostic prediction model developed from this subtyping system demonstrated stable predictive performance across multiple independent cohorts (AUC>0.6). The model not only accurately predicts patient prognosis but also provides crucial reference for immunotherapy benefit population screening and personalized treatment plan development. Notably, we found that low-risk group patients possess more active immune microenvironments and better immunotherapy responses, providing new evidence for clinical treatment decision-making. Drug sensitivity analysis further supports personalized treatment strategies based on risk stratification, providing a theoretical foundation for treatment selection across different risk groups.

## Data Availability

The data presented in the study are deposited in the Gene Expression Omnibus (GEO) repository, accession number GSE72094, GSE91061, and GSE135222, and the Cancer Genome Atlas (TCGA-LUAD) repository, accession number https://portal.gdc.cancer.gov/.
